# Exploring patients’ experiences with wAIHA and the content validity of the FACIT-fatigue: a qualitative interview study

**DOI:** 10.1186/s13023-025-03767-4

**Published:** 2025-08-06

**Authors:** Anubha Shukla, Owen Cooper, Karen Jones, Quentin A. Hill, Keith McCrae, Bruno Fattizzo, Michael Kostikas, Marieke Krol, Stephanie Bascle, Sylvie Bozzi, Ahmed Daak, Joshua Maher

**Affiliations:** 1 IQVIA, Bangalore, Karnataka India; 2 IQVIA, London, UK; 3 wAIHA Warriors, Marion, OH USA; 4 Leeds Teaching Hospitals NHS Trust, Leeds, UK; 5 The Cleveland Clinic, Cleveland, OH USA; 6 Università Degli Studi Di Milano-Bicocca, Milan, Italy; 7 IQVIA, Herikerbergweg 314, 1101 CT Amsterdam, The Netherlands; 8 Sanofi, Gentilly, France; 9 Sanofi, Cambridge, MA USA; 10 Sanofi, Reading, UK

**Keywords:** WAIHA, Anaemia, FACIT-Fatigue, Patient experience, Patient interviews, Content validity, Concept elicitation

## Abstract

**Background:**

Warm Autoimmune Hemolytic Anemia (wAIHA) is a rare form of anemia caused by premature destruction of red cells. This qualitative study aimed to understand the patient experience with wAIHA and provide evidence of FACIT-Fatigue scale validity by interviewing adult wAIHA patients along with examining patients’ definitions of meaningful change for item 1 of the FACIT Fatigue.

**Methods:**

Hybrid Concept Elicitation (CE) and Cognitive Debriefing (CD) interviews, lasting approximately 75 min, were conducted with US-based adults diagnosed with wAIHA. The CE part of the interview focused on understanding the patient's experiences with wAIHA, particularly the signs/symptoms, and impacts. The CD part evaluated patient’s feedback on clarity, relevance, and comprehensibility of the FACIT-Fatigue, and examined their interpretation of meaningful change for item 1.

**Results:**

Nineteen adult wAIHA patients (mean age 49.7 years) from the US were interviewed, 31 signs/symptoms were identified with fatigue being the most common (reported by all patients, 100%) and bothersome. In addition, many patients experienced shortness of breath (95%) and skin/eye color change (74%). Other common symptoms included headaches (63%), dark/brown urine (63%), heart palpitations (63%), and dizziness (53%). The study also found that wAIHA can have negative impact on patients’ emotional functioning, physical functioning, daily activities, social and professional life. The most frequently reported impacts included feeling anxious or scared (79%), difficulty climbing stairs (74%), needing help from others (74%), and reduced physical strength/mobility (74%). Patients also frequently reported feelings of anger/frustration (68%), needing frequent rests (68%), difficulty walking (68%), feeling worrisome (53%), and impacts on social activities (53%). A novel conceptual disease model was developed, highlighting the associations between symptoms and their impacts on patients’ lives. Patients indicated the FACIT-Fatigue was clear, relevant, and appropriate for measuring their wAIHA related fatigue. A 1-point score change in the item 1 was considered meaningful for both improvement and worsening of their fatigue by most patients.

**Conclusions:**

The study offers insight into the patient experience with wAIHA, detailing common symptoms and impacts on daily life as part of wAIHA conceptual disease model. It also supports the content validity of the FACIT-Fatigue instrument for assessing fatigue in wAIHA patients.

**Supplementary Information:**

The online version contains supplementary material available at 10.1186/s13023-025-03767-4.

## Background

Warm Autoimmune Hemolytic Anemia (wAIHA) is a rare type of anemia characterized by the premature destruction of healthy red blood cells (hemolysis), whereby red blood cells are “tagged” with auto-antibodies and are then destroyed by other types of immune cells [1]. wAIHA is the most common type of autoimmune hemolytic anemia; it remains relatively uncommon affecting approximately 1–3 per 100,000 people every year and can occur at any age [2]. The rarity of wAIHA poses challenges for diagnosis and treatment, often requiring specialized care and management strategies to address the unique needs of affected individuals [3]. The disease is termed “warm” because the antibodies are most active and cause hemolysis at body temperature, which is not necessarily the case in other types of autoimmune hemolytic anemia [4].

wAIHA may cause a variety of signs/symptoms which are bothersome to patients [5], Research with wAIHA patients has focused on one prominent symptom—fatigue, which has been reported to affect most, if not all, people with wAIHA [6, 7]. Although the extent to which fatigue impacts the lives of people with wAIHA has been researched in two studies [6, 7], the impacts associated with other signs and symptoms along with fatigue are not well investigated.

At present there is minimal research on clinical outcome assessments in wAIHA with input from the patients themselves. Hence, a qualitative research study was conducted with the objective to better understand the experiences of wAIHA patients, and to document the associated signs and symptoms, and their impacts on day-to-day life. The findings can be used to inform an approach to measuring patient-relevant outcomes in clinical trials.

Fatigue had already been highlighted as a relevant and important symptom for wAIHA patients in previous research, hence, the validity of a patient reported outcome (PRO) instrument, the FACIT-Fatigue, was also assessed in the interviews. The FACIT-Fatigue is a 13-item instrument, which can be used to measure fatigue severity and impact directly from patients. It was initially developed for use in oncology but since its development it has been widely used in non-oncology anemia [8–11]. A recent study used the FACIT-Fatigue in patients with cold agglutinin disease (CAD) [12, 13], a different sub-type of AIHA. However, there is limited evidence for relevance, clarity and comprehensiveness as well as interpretation of meaningful change in people with wAIHA.

Therefore, an additional aim of this study was to establish whether the FACIT-Fatigue is content valid (relevant, clear and comprehensive) [14] for measuring fatigue in future clinical trials among people with wAIHA, and to analyze how these patients interpret meaningful change of fatigue severity by using the FACIT-Fatigue item 1 as a proxy for overall fatigue severity.

## Methods

### Study design

To meet the study objectives, semi-structured qualitative patient interviews were conducted with adult patients diagnosed with wAIHA. Interviews included a concept elicitation (CE) portion to assess signs, symptoms, and impacts for wAIHA patients, along with a cognitive debriefing (CD) portion to assess content validity of the FACIT-Fatigue and examine patients’ interpretations of meaningful change for item 1 of the FACIT Fatigue (“I feel fatigued”). The study prioritized this item only for the evaluation of meaningful change, as patients may not be able to reliably estimate and provide their view on changes to the total instrument score.

### Study participants

To be eligible for the study, participants had to meet all of the following inclusion criteria—be 18 years or older at the time of consent, have a confirmed diagnosis of primary wAIHA or SLE-associated wAIHA (excluding other SLE symptoms except for skin and muscle involvement), be able to give signed consent, be able to complete a 75-min interview in English, and live in the US, excluding Puerto Rico.

The study recruited 19 patients from December 2022 to October 2023 through the patient recruitment agency Global Perspectives (an IQVIA business) and on social media, and in collaboration with wAIHA Warriors, a patient advocacy group. Global Perspectives informed and screened patients for eligibility. Participants provided consent and confirmation of their wAIHA diagnosis, and upon verification, were interviewed by IQVIA. Each participant received honorarium for their involvement, as determined by industry Fair Market Value rates. Patient confidentiality was ensured through researchers only having access to personal data on a need-to-know basis.

### Interview procedure

The IRB-approved semi-structured interview guide was designed according to study objectives. Mock interviews were performed to evaluate the guide and discuss the logic and flow of interview questions and probes. Following patient recruitment and scheduling, telephone interviews lasting approximately 75 min each were conducted by two experienced IQVIA moderators, trained in CE and CD patient interview methodology. Interviews were open-ended to encourage a natural, free-flowing conversation between the interviewer and participant, and the process adhered to International Society of Pharmacoeconomics and Outcomes Research (ISPOR) Good Research Practices Task Force guidelines [14, 15]. Audio files were transcribed verbatim for analysis, and transcripts were anonymized.

#### Concept elicitation (CE)

During the CE portion of the interview, wAIHA patients were asked open-ended questions to encourage participants to share, in their own words, their experiences with wAIHA. Participants discussed the signs, symptoms, and daily impacts of their wAIHA both spontaneously and after being probed. Concepts were scored as “spontaneous” if the patient mentioned the concept unaided by the moderator, and as “probed” if the moderator asked about the concept directly and the patient confirmed that they had experienced this concept. Examples of starter questions which were asked to the patients, and from which discussion was facilitated included:

- What symptoms do you typically experience due to [patient’s terminology for wAIHA]?
- Do you have any impacts to your life due to [patient’s terminology for wAIHA]? If so, please explain.
- Are you currently on or have you previously been on medication for [patient’s terminology for wAIHA]?

Patients were asked to report the top three most bothersome concepts of their condition and rated the bothersomeness of each sign, symptom or impact on a scale from 0 to 10, with 0 being not bothersome at all and 10 being as bothersome as the patient can imagine. For the signs and symptoms, bothersomeness rating was asked for two timepoints—at the time of the interview (current) and at its worst (at diagnosis/flare-up) to identify the difference in ratings at these time points. For impacts, a bothersomeness rating was provided only reflecting their “current” bothersomeness. Sample questions which were initially asked to the patients included:

- From the symptoms I have read out loud [that the patient reported], which 3 symptoms are the most bothersome to you? Why?
- On a scale of 0 to 10 can you please rate how bothersome each of these symptoms are to you?

#### Cognitive debriefing (CD)

Participants were presented FACIT-Fatigue items through a web-based screen-sharing application (Mercuri) and were instructed to read and answer the items aloud. The purpose of the CD was to evaluate the comprehension and relevance of the items, response options (*Not at all/A little bit/Somewhat/Quite a bit/Very much*) and recall periods (*past 7 days*) of FACIT-Fatigue for wAIHA patients. Since the instrument’s recall period and response options are identical for all items, their relevance and comprehension were only assessed once, during the evaluation of item 1. Sample questions which were asked to the patients included:

- How would you answer this question? Why did you pick your answer? Do you feel this item is important to your experience of wAIHA?
- Do you find this item unclear or confusing in any way?
- Let’s look at the response options “not at all” through to “very much”, what do each of the categories mean to you? Are they clear to you as they are worded, or do you find them to be unclear or confusing?
- Is it easy to recall your symptoms over “the past 7 days”? Explain why or why not.

Furthermore, participants were requested to share their opinions on what they would consider a meaningful change (meaningful improvement and meaningful worsening) in the FACIT-Fatigue item 1, both quantitatively (using the response scale) and qualitatively (in terms of how their lives would change with a meaningful improvement or deterioration in their wAIHA).

### Data analysis

Descriptive statistics were used to analyze the demographics and clinical characteristics of the sample.

A qualitative analysis plan (QAP) was developed before data collection which was executed during transcript coding and data analysis. A preliminary codebook was created as part of this QAP. This codebook was updated throughout the study in light of the information provided throughout the interviews. These codes were applied to de-identified transcripts following each interview wave by a primary and secondary researcher using MAXQDA software [16] to ensure consistent data analysis. The primary coder analyzed all transcripts, while the secondary researcher reviewed 20% of them, with regular meetings and quality checks to ensure accuracy.

Patient reported concepts were integrated into a conceptual disease model. The study team utilized MAXQDA for exporting patient descriptions to Excel and analyzed them to merge overlapping concepts, divide complex ones into sub-components, exclude non-unique or vague concepts, and reword them into patient-friendly language, guided by the patients'terminology.

Saturation of concepts is defined as the point when no new relevant or important information emerges and collecting additional data will not add further to the patient experience understanding [17]. Saturation was evaluated in waves of five interviews each, with the final wave comprising four interviews. After each wave of interviews, assessments were conducted on whether any new concepts were identified. The assessment continued until no new concepts emerged, indicating that additional interviews would not add to, nor enhance the concepts already identified. Saturation tables from the entire sample were used to track concept saturation throughout the interview process. [18].

The mean bothersomeness rating was calculated for each sign, symptom, and impact that received patient ratings. When patients reported multiple bothersomeness ratings for a single symptom or impact, such as ‘a 6 or 7’, the highest rating was used for analysis.

A concept was considered frequently reported if 50% or more of respondents identified it as relevant based on their experience of the disease, either through probing or spontaneously. Similarly, a concept was classified as bothersome if it received an average (or mean) bothersomeness rating of 5 or higher on a 0–10 scale. The 50% threshold for relevance indicates that the concept is significant to a majority of respondents. Likewise, a bothersomeness rating of 5 or higher highlights concepts that are moderately to highly bothersome. Therefore, this criterion for identifying ‘frequent and bothersome’ concepts was used to determine key concepts of interest for the interviewed wAIHA population.

In the CD portion, patient understanding and relevance of the FACIT-Fatigue was also assessed through coding of transcripts. The ‘understanding’ code was used when patients found the wording clear or interpreted the items as intended. The ‘response option clarity’ code was applied when patients understood and could relate to the response options. The relevance of items and response options was coded based on patient statements about their appropriateness and relevance to their experience.

For meaningful change data, participants’ ‘current’ responses, the minimum change in item score that would be meaningful, and the impact of such changes on daily life were coded. Data from FACIT-Fatigue Item 1 was collected, with participants discussing what comprises a meaningful level of change for their wAIHA experience. Responses across participants were summed and/or averaged and the total, mean, standard deviation, and range were reported. Qualitative descriptors of how their lives would change with a meaningful improvement or worsening were coded and synthesized.

## Results

### Patient demographics and disease journey

The study involved interviews with 19 adult patients residing in the US. Their socio-demographic information is detailed in Table 1. The average participant age was 49.7 years old. Most participants were white (79%), with 4-year college degree or higher (95%) and were females (63%).

**Table 1:** Socio-demographic details of the patient sample (N = 19)

Demographic	n	Percentage
Age (years)		
Mean (SD)	49.7 (14.2)	–
Median	48	–
Gender		
Female	12	63%
Male	7	37%
Highest level of education completed		
Some college	1	5%
Completed 4-year college degree	12	63%
Graduate college degree or higher (e.g., master’s degree, PhD)	6	32%
Race/Ethnicity		
Asian/Pacific Islander	1	5%
Hispanic/Latino	2	11%
White (Non-Hispanic/Latino)	15	79%
Mixed (Black and White)	1	5%

Table 2 presents the clinical profiles of these patients, including diagnosis timeframes and treatments. The median time since diagnosis was four years (range: 1–13 years) with the majority of patients being diagnosed with wAIHA within the past six years. 32% were using steroids, 16% rituximab, and 11% other treatments at time of screening.

**Table 2:** Clinical details of the patient sample (N = 19)

Diagnosis	n	Percentage
Time since diagnosis (months)		
Mean (SD)	64.9 (48.37)	–
Median	49	–
wAIHA type		
Primary wAIHA	17	89%
Systemic lupus erythematosus (SLE)-associated wAIHA	2	11%
Time Since Diagnosis Ranges		
0 ≤ 3 years ago	6	32%
3 ≤ 6 years ago	7	37%
6 ≤ 9 years ago	2	11%
9 ≤ 12 years ago	2	11%
12 ≤ 15 years ago	2	11%
Current treatments received for wAIHA		
Steroids	6	32%
Rituximab	3	16%
Other	2	11%
Not specified during interview	10	53%
Previous treatments received for wAIHA		
Steroids	15	79%
Rituximab	13	68%
Splenectomy	5	26%
Blood transfusions	5	26%
Other	6	32%
Not mentioned	1	5%

Patients shared their experiences leading to a diagnosis of wAIHA. Most (95%) consulted doctors due to symptoms, including fatigue, difficulty breathing, jaundice, and rapid heartbeat, often in combination, thus leading to a diagnosis by hematologists following tests such as the Coombs test. Four patients (21%) were initially misdiagnosed, six (32%) received a quick diagnosis (via “pretty immediate” blood test) and seven (37%) received a delayed diagnosis (blood test indicated low hemoglobin but wAIHA was confirmed in follow up tests conducted couple of days later).

Three patients reported relapses in their condition; one experienced a relapse six year post-diagnosis following a COVID-19 infection, another relapsed nearly a year after their initial diagnosis, and the third relapsed three years into remission. Additionally, three patients were in remission during the interview.

### Experiences of wAIHA patients

#### Signs/symptoms

**Fig. 1:**
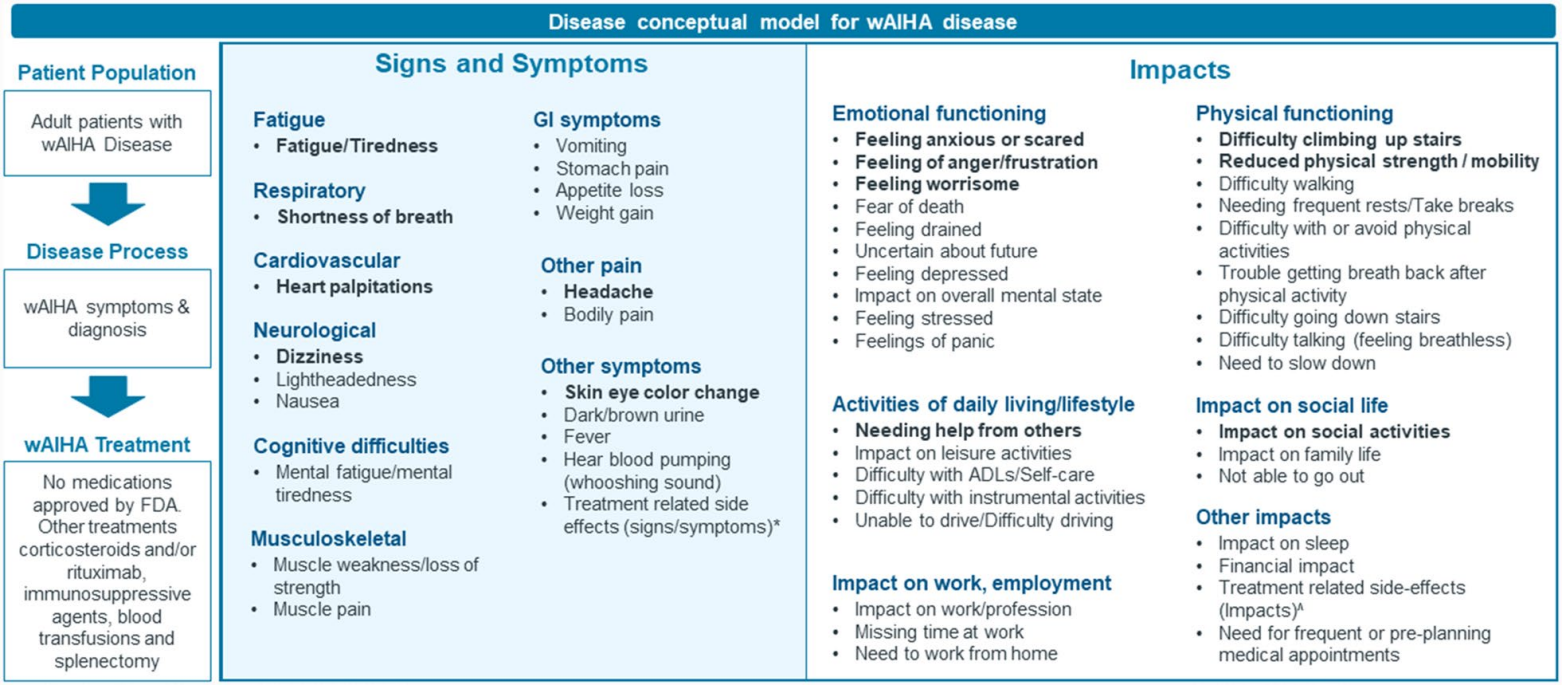
Conceptual disease model of adult wAIHA patient experience. Bold font: Frequent and bothersome signs/symptoms or impacts defined as ≥ 50% of respondents reporting the concept as relevant (either probed or spontaneous) with a mean bothersomeness rating of ≥ 5 on a scale from 0 to 10; Abbreviations: ADL—Activities of daily living, GI – Gastrointestinal, FDA—Food and Drug Administration, wAIHA—Warm antibody autoimmune hemolytic anemia; *Treatment related side effects (signs/symptoms) included side effects to steroid use (prednisone), such as pneumonia, diabetes, increased appetite, weight gain, hallucinations, wheezing, cough, fatigue, frequent urination, joint pain, lightheadedness, dizziness, headache, shortness of breath, and stomach pain. Side effects related to Rituximab were also reported. These included pneumonia, joint pain, nausea, wheezing, cough, fatigue, body swelling, hair loss, frequent urination, shortness of breath, and muscle weakness; ^Treatment related side effects (impacts) were associated with steroid use (prednisone), causing mood swings and sleep disturbances. Depression was noted as a side effect of lengthy infusions. Additionally, pain medications were reported to cause mood swings; NOTE: Only signs, symptoms and impacts reported by 2 or more patients are shown. Other symptoms reported by n = 1 patients: Vision loss, Night sweats, Chills/Feeling cold, Loss of consciousness, under eye dark circles, thinning of nails, Constipation, Acid reflux, Weight loss, Pounding in head, and Low blood pressure. Other impacts reported by n = 1 patients: Emotional impact (lack of motivation, embarrassment, feeling guilt), Other impacts (medical anxiety/distrust, impact on sexual activity, impact on quality of life, not able to live life fully, feeling misunderstood, and impact on diet

Thirty-one signs/symptoms were reported by patients during the interviews. The complete list of identified signs and symptoms is listed in Fig. 1. Saturation was achieved for signs/symptoms with no new important concept emerging in the last wave of patient interviews.

All patients experienced fatigue (100%), with the majority also reporting shortness of breath (95%) and skin/eye color changes (74%). Other common symptoms included headache (63%), dark/brown urine (63%), heart palpitations (63%), and dizziness (53%). Over a quarter of patients reported mental fatigue/tiredness (42%), nausea (37%), appetite loss (37%), lightheadedness (32%), and side effects from treatment (32%).

The study revealed that fatigue (95%) was the most bothersome symptom, affecting daily life and work—*“In my mind, it’s the fatigue. Because that’s what keeps me from doing stuff.”* Shortness of breath (47%) and heart palpitations (42%) were also commonly reported as bothersome, impacting daily activities, sleep, and fear. Other symptoms were also noted, but infrequently. Supplementary Table 1 provides patient quotations for the top most bothersome signs/symptoms.

**Fig. 2:**
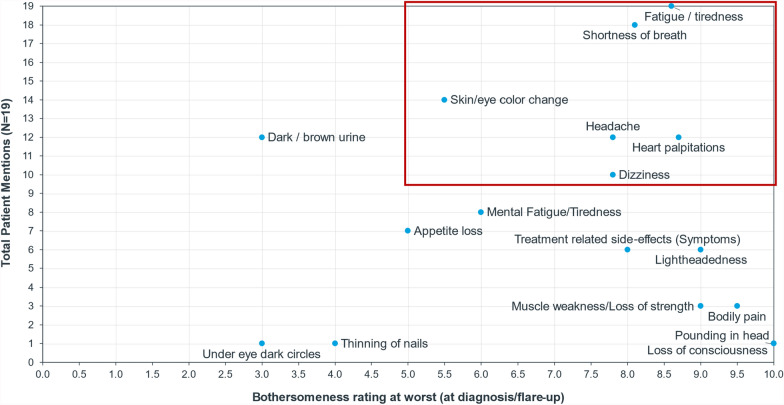
Signs/symptoms based on bothersomeness rating at its worst (at diagnosis/flare-up). Frequent and bothersome signs/symptoms are highlighted in red outlined box

Figure 2 graphically presents the frequency of signs/symptoms and average bothersomeness rating at the time of the interview and at its worst (diagnosis/flare-up). Fatigue was the only symptom meeting the criteria of most frequent and bothersome based on the bothersomeness rating at the time of the interview. At the worst point, additional symptoms such as shortness of breath, skin/eye color change, headache, heart palpitations, and dizziness were also considered frequent and bothersome based on the pre-defined cutoff. These symptoms, while not always present, can be significantly bothersome when they occur, with fatigue being consistently prominent and bothersome.

Table 3 contains patient quotations for frequent and bothersome signs/symptoms, while Supplementary Table 2 contains quotes for other signs/symptoms.

**Table 3:** Patient quotation associated with the frequent and bothersome signs/symptoms

Signs/symptoms**	N = 19 [n(%)]	Mean bothersome rating at worst (at diagnosis/flare-up) * [n, rating range]	Patient quotations
Fatigue/tiredness	19 (100%)	8.6 (n = 15; 5–10)	“When it’s in full force, you’re debilitated. You can do things, you’re just very slow and you’re very, very sluggish.” “You know, if you’re tired, you’re not going to want to go out and do any activities because you’re too tired. You don’t care what it is.”
Shortness of breath	18 (95%)	8.1 (n = 9; 3–10)	“When my blood levels would go to a very low, dangerous point, and my shortness of breath was at its worst, I could barely tie my shoes without being out of breath. I couldn’t make my bed. Cooking was even…it took an effort. It was as if I was grasping for air.” “I noticed walking upstairs or walking up a hill. I was like, man, I’m really getting out of shape. I need to do something about this. Then, as it progressed over time, it just… Just walking, just down the hallway, I’d be like, phew, kind of out of breath. Again, it just got increasingly worse. To the point just walking around the house.”
Skin/eye color change	14 (74%)	5.5 (n = 2; 3–8)	“Then I got jaundice, so I was completely turning yellow.” “My eyelids were really white. I was really pale”
Heart palpitations	12 (63%)	8.7 (n = 7; 5–10)	“You know what, that is one major thing I did forget to mention earlier as a symptom. Yes, I have a very high heart rate almost all the time. I can’t believe I forgot that one.” “My heart rate was through the roof. […] So when I'm having a flare-up, I can tell if I get up and walk to a different room and sit down, and I can just feel my heartbeat in my chest.”
Headache	12 (63%)	7.8 (n = 6; 4–10)	“Just constant headache. It felt like it was mainly in my eyes and sinuses.” “Then my headache started getting… There’s a name for it. I don’t know the name for it. But it was like a headache I’ve never experienced before. It was more like a jackhammer going off in my head. Like a pounding in my head that I just could not get away from.”
Dizziness	10 (53%)	7.8 (n = 5; 6–10)	“I get dizzy spells…That’s more like when I stand up, when I’ve been sitting down or laying down, and I first get up, I get real dizzy. I have to hold onto something for a minute. Then it goes away and then I’m okay.” “Started…when I would stand up, I’d be dizzy, disoriented, falling over.”

* Bothersomeness ratings were assessed on a 0-10 scale, where 0 = not bothersome at all and 10 = as bothersome as you can imagine. Average Bothersomeness ratings are based on the number of patients who provided a rating which is not always the same as the number of patients who endorsed the symptom. Some patients provided qualitative descriptions and even with gentle encouragement by the moderator would not provide a quantitative number. ** Only symptoms reported by 10 or more patients are shown in the Table

#### Impacts

Forty-two impacts were reported by the patients during the interviews which were categorized into six different impact categories. This included emotional functioning (13 impacts), physical functioning (9 impacts), ADLs/lifestyle (5 impacts), social functioning (3 impacts), professional impacts (3 impacts), and other impacts (9 impacts). The complete list of impacts is listed in Fig. 1. Saturation was achieved for impacts with no new important concept emerging in the last wave of patient interviews.

The most frequently reported impacts were feeling anxious or scared (79%) followed by difficulty climbing upstairs (74%), needing help from others (74%), and reduced physical strength/mobility (74%). Patients also frequently reported feeling of anger/frustration (68%), needing frequent rests or take breaks (68%), difficulty walking (68%), feeling worrisome (53%), and impact on social activities (53%).

All patients identified at least one bothersome impact, with five patients (26%) reporting feeling anxious or scared as their primary concern related to the unpredictability of their condition, including concerns about future relapses and treatment effectiveness. Four patients (21%) found needing help from others particularly bothersome due to the perceived burden on others. Other impacts were also noted as bothersome, albeit infrequently. Supplementary Table 1 provides patient quotations related to the most bothersome reported impacts.

**Fig. 3:**
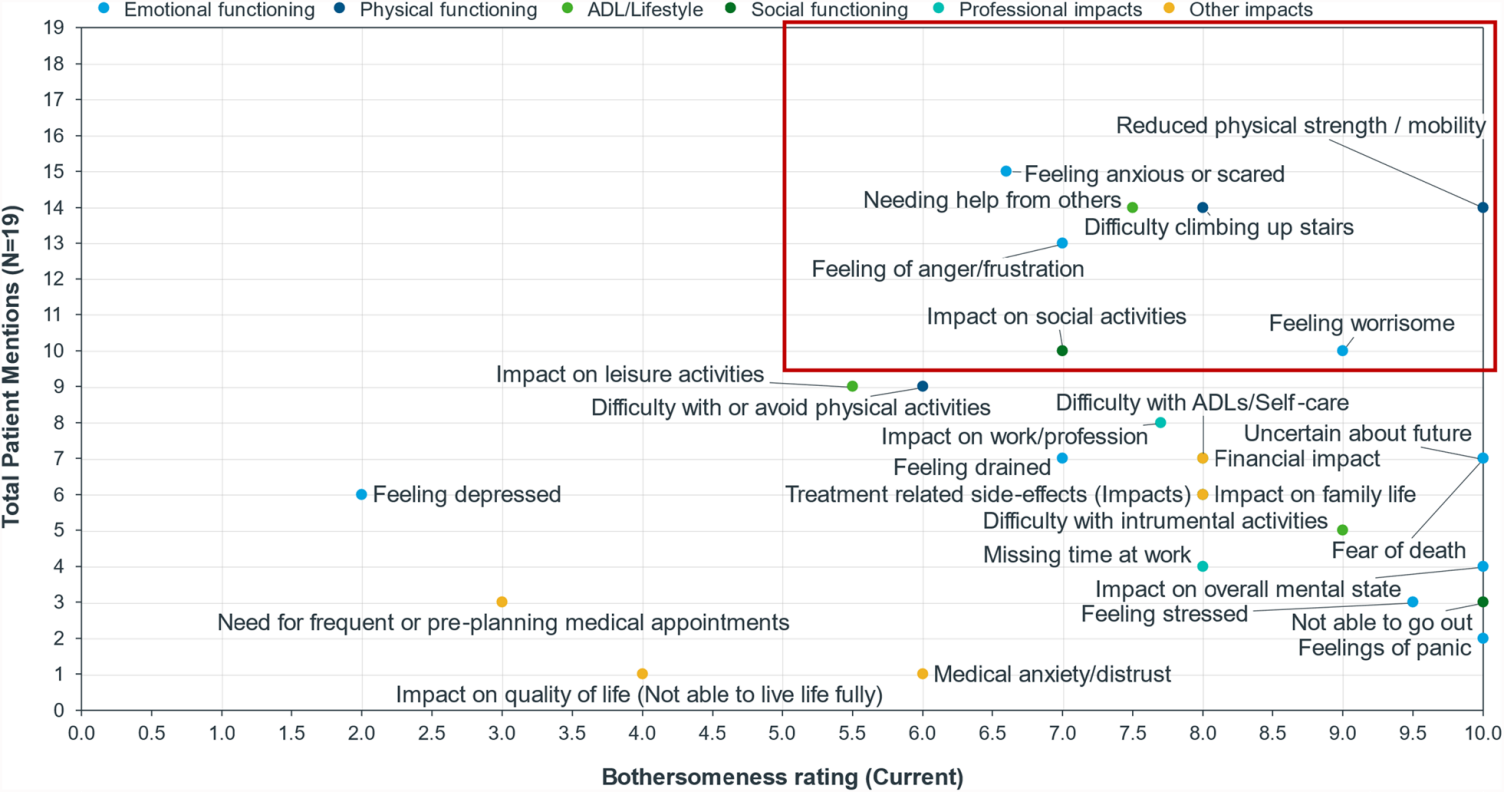
Impacts based on bothersomeness rating. Frequent and bothersome impacts are highlighted in red outlined box

Figure 3 graphically presents the impacts experienced by patients by frequency and how bothersome these impacts were rated at the time of the interview. Frequent and bothersome impacts included feeling anxious or scared, reduced physical strength/mobility, difficulty climbing upstairs, feeling of anger/frustration, needing help from others, feeling worrisome, and impact on social activities.

Table 4 contains patient quotations for the frequent and bothersome impacts, while Supplementary Table 3 provides quotes for other mentioned impacts.

**Table 4:** Patient quotation associated with the frequent and bothersome impacts

Impacts**	N = 19 [n(%)]	Mean bothersome rating (current)*	Patient quotations
*Emotional impact*			
Feeling anxious or scared	15 (79%)	6.6 (n = 5; 3–9)	“It gives me anxiety to not know why, like what triggered it or to not know what triggered the relapse. So I feel anxious about having another relapse. When is it going to happen? Is it going to happen? What if the treatments don’t work this time? Kind of thing. Kind of the unknown, I guess, is a little scary, because there’s not a lot of people to talk to that have experienced it.” “I feel chronic anxiety from my condition. I mean, that's one of the major effects it's had on my life. It makes me more… I mean, it's kind of like proof, right? There's leukemia in my family, so it makes me worry that I'm susceptible to like a blood cancer.”
Feeling of anger/frustration	13 (68%)	7.0 (n = 1)	“Just having to deal with this. I don't feel sad for me. I'm just pissed off. Just one more of those interesting life experiences I got to deal with. I thought I'd been through enough of them in my life.” “I have been going to the doctor pretty much every week since the end of January, and that’s a lot of co-pays from the insurance. And that right there gets to be very irritating.”
Feeling worrisome	10 (53%)	9.0 (n = 1)	“But also it can be to the point where you get very worried about it, like why is this happening.” “Yeah, there definitely was a lot of that because of the unknown. Am I going to get better?”
*Physical functioning*			
Reduced physical strength/mobility	14 (74%)	10.0 (n = 2; 10–10)	“I couldn’t take out the trash. I’d break out in sweat. I could, it just take me forever. It would, literally just to function.” “I sometimes still feel like I lack strength. I lack the ability to do certain things.”
Difficulty climbing up stairs	14 (74%)	8.0 (n = 1)	“I tend to climb stairs to give myself exercise, and so 3 stories…by the time I got on top of 3 stories I’d be like seeing stars.” “Just at first, I noticed walking up stairs or walking up a hill. I was like, man, I’m really getting out of shape. I need to do something about this.”
*Activities of daily living/Lifestyle*			
Needing help from others	14 (74%)	7.5 (n = 4; 6–9)	“The general weakness of limiting any activities around the house I might want to do, minor repairs, maintenance.” “I couldn’t vacuum. I wouldn’t dust. I couldn’t take out the trash. I’d break out in sweat. I could, it just take me forever. It would, literally just to function. I’m not going to beat myself up, my husband ends up doing most of everything literally.”
Social impacts			
Impact on social activities	10 (53%)	7.0 (n = 2; 5–9)	“Yeah, because I’m the type of person… As soon as work’s done, I was either at the gym or off doing a social activity. I just couldn’t do it.” “That’s cute that you think I have a social life. I don’t feel like I have a social life. But I’m going to say…I would say yes because…You know, if you’re tired, you’re not going to want to go out and do any activities because you’re too tired. You don’t care what it is.” “I’m too tired to do anything with my friends, so that’s the symptom that keeps me from going out.”

* Bothersomeness ratings were assessed on a 0-10 scale, where 0 = not bothersome at all and 10 = as bothersome as you can imagine. Average Bothersomeness ratings are based on the number of patients who provided a rating which is not always the same as the number of patients who endorsed the impact. Some patients provided qualitative descriptions and even with gentle encouragement by the moderator would not provide a quantitative number. ** Only impacts reported by 10 or more patients are shown in the Table

### Conceptual model

**Fig. 4:**
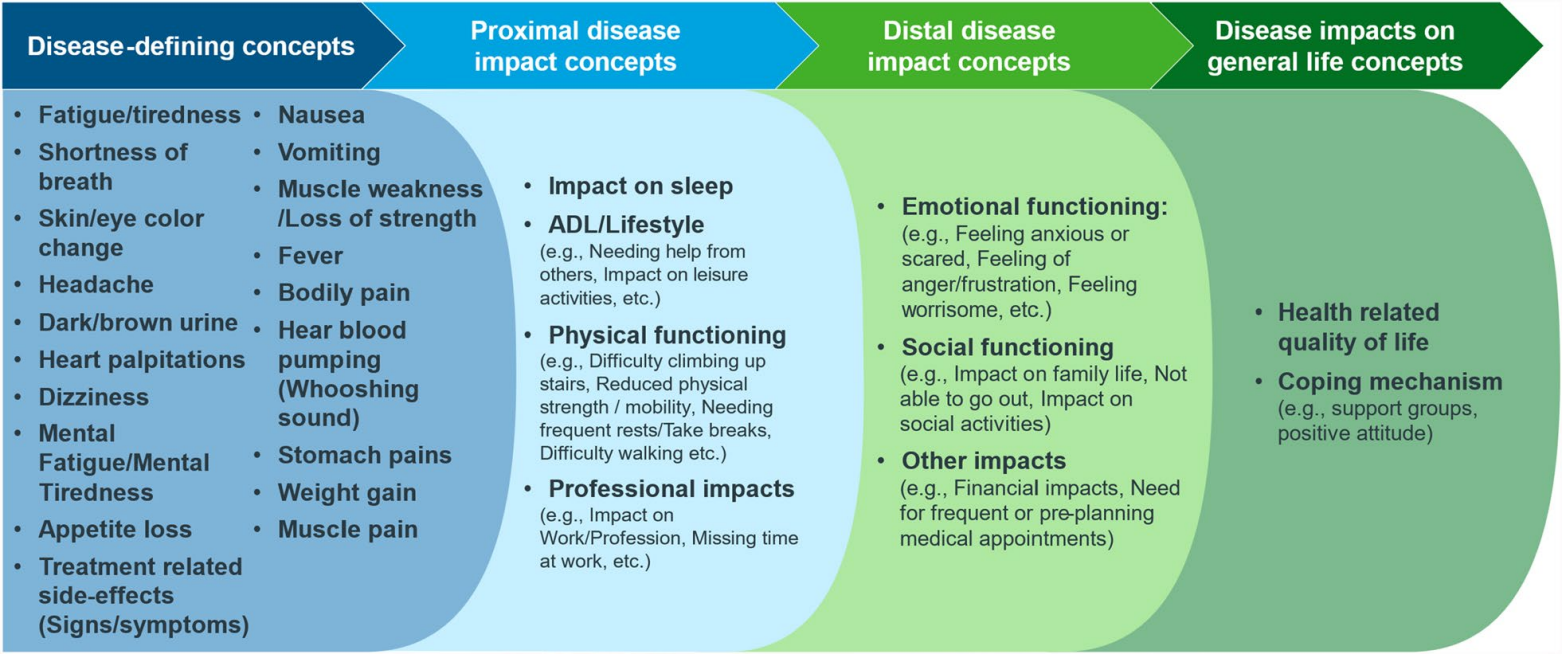
Hypothesized proximal–distal continuum between identified signs, symptoms & impacts

The patient experience data generated from these interviews were used to create a novel conceptual disease model of wAIHA in adults (Fig. 1). Figure 4 shows the impacts on a hypothesized proximal–distal continuum based on the qualitative research findings and general understanding of quality of life (as per the Wilson and Cleary model [19]).

**Fig. 5:**
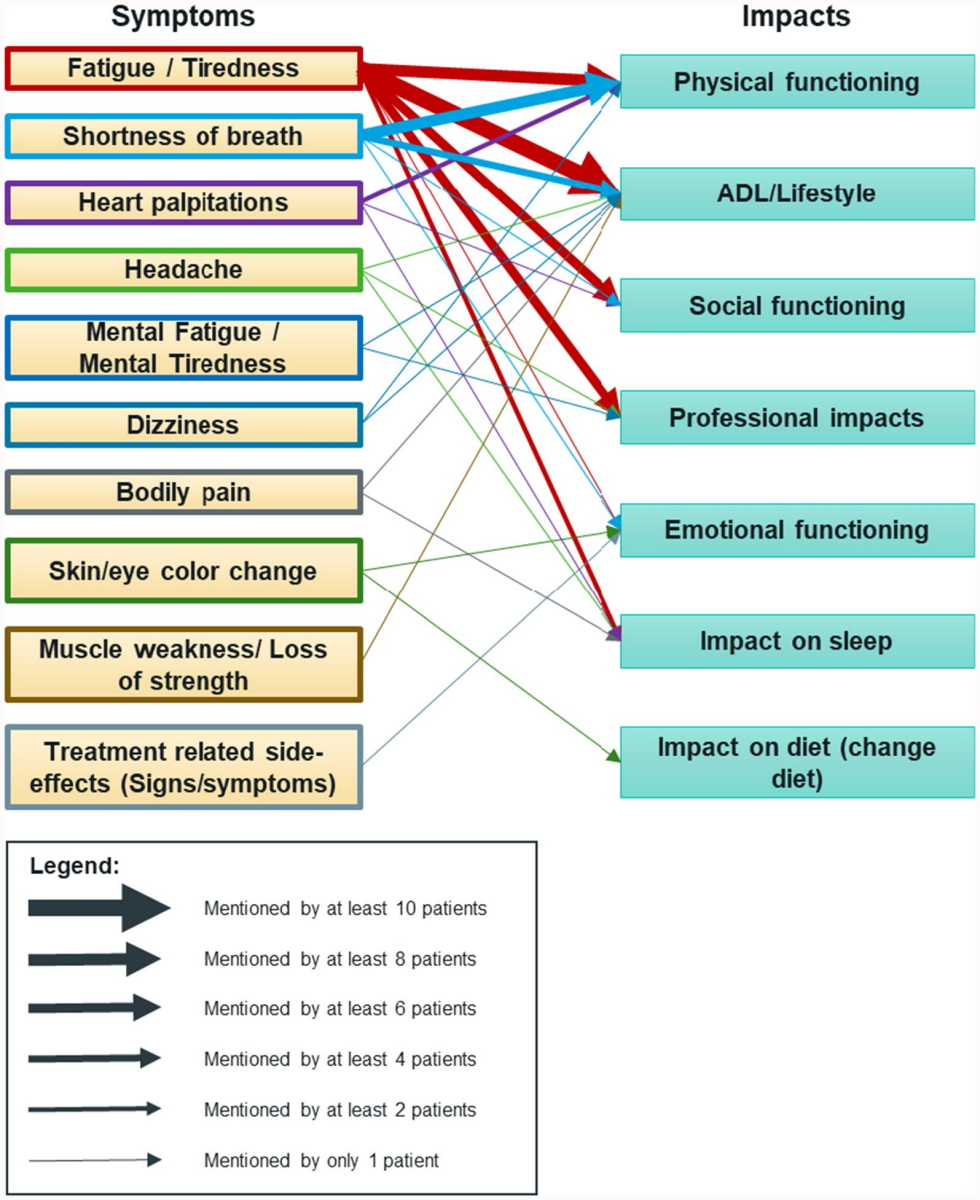
Causal and consequential relationship between identified signs, symptoms & impacts, as mentioned by the patients during the interviews. Note: The color-coded outline on the boxes that represent the symptoms is designed to assist in identifying which arrow corresponds to each symptom

Some patients also detailed the associations between their signs/symptoms and impacts they experienced. These associations are captured in Fig. 5. Fatigue or tiredness impacts all six impact categories identified during patient interviews: ADL/lifestyle, physical functioning, social functioning, professional impacts, emotional functioning, and other impacts. Shortness of breath also influences physical functioning, ADL/lifestyle, social functioning, and emotional functioning.

### Content validity of FACIT-Fatigue

The FACIT-Fatigue questionnaire was completed by all patients, who provided feedback on instructions, items, and response options. All patients understood the instructions, with one suggesting a minor modification for clarity. All patients found the items relevant to their experience with wAIHA and were able to provide a response to all of the items. However, one patient struggled with item 3 (“I feel listless (washed out)”), while six patients found item 7 (“I have energy”) challenging due to its perceived ambiguous phrasing and lack of detail regarding whether it is related to physical or mental state. The patients also noted that item 7 overlaps with other items. Most patients answered ‘a little bit’ or ‘not at all’ for all items, except for item 7 and item 8 (“I am able to do my usual activities”), which showed that the patients had their fatigue controlled at the time of the interview.

A summary of patient feedback is detailed in Supplementary Table 4. Overall, the patients found the response options clear. Patients also explained how they interpreted each of the response options for the FACIT-Fatigue. A summary of patient feedback of the response options is detailed in Supplementary Table 5. All patients who were asked found the recall period acceptable and felt they were able to remember this time frame to answer questions accurately. Feedback from two patients included a suggestion to extend the timeframe for some items as *“there wasn’t anything in the last 7 days”* and a recommendation to add *‘during an episode’* for those in remission.

### Meaningful change FACIT-Fatigue item 1

Patients were asked what improvement they would need to see from their selected response in the FACIT-Fatigue item 1 in order for the change to be an important meaningful change for them in their wAIHA-related fatigue. Conversely, they were also asked what worsening they would need to see for it to have a meaningful negative impact. The majority of patients (n = 15) noted that 1-point improvement in FACIT-Fatigue item 1 would be meaningful to them, while the remainder (n = 4) indicated they would consider a 2-point change meaningful. Similarly, for meaningful worsening overall, the majority of patients (n = 13) indicated that 1-point worsening would be meaningful for FACIT-Fatigue item 1, while relatively few patients for FACIT-Fatigue item 1 (n = 3) considered a 2-point change meaningful. Three patients were not asked about meaningful worsening due to lack of time. No patients proposed a 3 or 4-point changes in order to consider the worsening meaningful. Participants described that a meaningful improvement in their symptoms would be one which have a significant impact on their overall mental and physical wellbeing or the activities they would be able to do.

*“When I think “very much,” that's when I am just taking naps all the time to where “quite a bit” is where I'm actually going in the grocery store.”*

Patient responses regarding meaningful improvement and worsening for FACIT-Fatigue Item 1 by individual item response is shown in Supplementary Table 6. The means, standard deviations, and ranges of patients’ overall responses for meaningful change are listed in Table 5.

**Table 5:** Mean, standard deviation and range on the patients’ assessment of perceived meaningful improvement and worsening of the FACIT-Fatigue Item 1

	Improvement				Worsening			
	N	Mean	Standard deviation	Range	N	Mean	Standard deviation	Range
FACIT-Fatigue item 1	19	− 1.21	0.4	− 2 to − 1	16	1.19	0.4	1–2

## Discussion

Warm Autoimmune Hemolytic Anemia (wAIHA) is indeed a rare and life-threatening disorder, with an incidence of approximately 1–3 cases per 100,000 people annually [1, 2]. This rarity poses significant challenges in both diagnosis and treatment, often requiring specialized care. Despite being the most common form of autoimmune hemolytic anemia, wAIHA remains under-researched, with few advancements in treatment over the past decades. Recent years have seen a surge in research interest, leading to the development of potential new therapies [20].

This study aimed to understand patients’ experiences with wAIHA and their perspective on a patient reported outcome instrument, the FACIT-Fatigue, through combined qualitative CE and CD interviews with adult wAIHA patients.

The CE portion of the interviews focused on understanding the key signs, symptoms, and impacts of wAIHA, identifying the most frequent and bothersome concepts to patients. Key findings include fatigue, shortness of breath, skin/eye color changes, headaches, heart palpitations, and dizziness as frequent and bothersome symptoms, with physical fatigue being the most critical, consistent with prior research [6, 7, 21, 22]. Patients described feeling exhausted, which was not improved after resting or sleeping. Many experienced constant fatigue during flare-ups, impacting their ability to perform tasks and requiring greater effort and a slower pace. Shortness of breath was another prevalent symptom causing difficulty in breathing, and making physical activities and household chores challenging. Patients often felt anxious and fearful, particularly when shortness of breath occurred unexpectedly or during minimal exertion. For many patients, the combination of shortness of breath and heart palpitations further exacerbated these feelings, making it challenging to carry out even simple tasks. Several patients reported changes in skin and eye color, causing anxiety due to visibility. The yellowing served as a constant reminder of their condition, affecting self-esteem and social interactions, and contributing to the emotional burden. Headaches were common, with varying severity. Some experienced mild annoyance, while others found them debilitating, preventing daily activities. Persistent pain often required medication, providing only temporary relief. Heart palpitations, characterized by rapid heart rate and pounding sensation, led to fear and concern. This symptom often co-occurred with shortness of breath, compounding the burden and making daily life difficult. Patients often had to slow down or rest to alleviate this symptom. Shortness of breath as part of the fatigue, and the combination of shortness of breath and heart palpitation with fatigue were also reported by one patient each. These combinations intensified the overall impact on patients, making it more challenging to cope with their condition.

The study also highlighted the various impacts of wAIHA, which were categorized into emotional functioning, physical functioning, activities of daily living (ADLs)/lifestyle, social functioning, professional impacts, and other impacts. Emotional impacts, such as anxiety and fear, were particularly prominent, reflecting the psychological burden of living with a chronic condition. The uncertainty about future relapses and treatment effectiveness contributed to these emotional challenges. Physical limitations, including difficulty climbing stairs, reduced physical strength/mobility, and the need for assistance from others, were frequently reported. These limitations affected patients' independence and quality of life. The study also revealed the social and professional impacts of wAIHA, with patients reporting difficulties in participating in social activities and maintaining their work responsibilities. Patients identified feeling anxious or scared, needing help from others, and impact on work as the most bothersome impacts due to wAIHA. These concerns were primarily driven by the unpredictability of the condition, the burden on caregivers, and the inability to perform tasks effectively. These impacts reflect the broad and pervasive nature of the disease’s impact on patients' lives.

A novel wAIHA disease conceptual model for adult patients was developed. Additionally, the research noted the association between symptoms and their effects on various aspects of life, with fatigue impacting the broad range of categories. These associations were not probed for all patients due to time constraints; however, they provide valuable insights into the patient experience of wAIHA.

The CD portion of the interviews focused on the FACIT-Fatigue. The FACIT-fatigue is an instrument that can be used to measure fatigue in many health conditions. The interviews aimed to verify its relevance and understandability specifically among wAIHA patients. The questionnaire was found to be relevant, comprehensible, and had an acceptable recall period to patients, indicating its content validity for wAIHA patients. These findings in wAIHA are consistent with findings supporting the content validity of the FACIT-Fatigue in other patient groups [8, 23].

The qualitative assessment on meaningful change, conducted a posteriori*,* revealed that most patients in the interview study deemed a 1-point change on the FACIT-Fatigue item 1 as meaningful to them, aligning with a prior qualitative threshold estimate [6]. While this insight can aid in interpreting the impact of study treatments on fatigue in wAIHA, meaningful change was only examined hypothetically and qualitatively in this study, and is not based on actual experiences of changes in patient disease status or the result of psychometric analysis. Although this approach is common and has been shown to produce reliable results [24], psychometric data about real changes perceived as meaningful are necessary to confirm this threshold. Additionally, this study does not provide insight into what constitutes a meaningful change in the FACIT-Fatigue total score as the identification of meaningful change was confined to item 1 of the FACIT-Fatigue. This limitation arises because meaningful change for the total score would need to be assessed at each individual item level. Due to the time constraints of the interviews, there was insufficient time to thoroughly examine meaningful change on each individual item of the FACIT-Fatigue. Consequently, item 1, “I feel fatigued”, was selected as it was deemed most representative of the PRO’s aim, given that the FACIT-Fatigue specifically measures fatigue.

In addition to fatigue, this study highlighted other symptoms such as shortness of breath and heart palpitations, which were relevant to patients but have not been focused on in previous wAIHA clinical trials or research. The data indicated a need to include a broader range of measurements for future assessment of fatigue, to capture the full spectrum of symptoms of the condition, as the FACIT-Fatigue questionnaire does not adequately reflect all relevant patient experiences in wAIHA as a whole, including mental fatigue and the emotional impacts associated with fatigue. Alternative PROs such as generic outcome measures (e.g., SF-12 [25], SF-36 [26]), or dyspnea-specific PROs may be considered to provide a more comprehensive understanding of the signs, symptoms and impact of wAIHA.

The study has some important limitations, including a gender imbalance in the sample, with more female participants than males, and participants only recruited in the US. This reflects a common issue in qualitative research, where male participation is typically lower [27, 28]. In addition, most participants were well-educated, which may influence their descriptions of symptoms and impacts, and their comprehension of the FACIT-Fatigue measure. These limitations limit the generalizability to the wAIHA population as a whole. However, due to how rare the condition is and the associated difficulties in recruiting patients to be interviewed for the study, recruiting such an ideal sample was not possible and is not uncommon with orphan status disease recruitment. Furthermore, due to the interview time constraints, it was not possible to explore the extent to which the impact of the disease on daily life remained after recovery or remission. This limitation highlights the need for future research to investigate the long-term effects of wAIHA on patients' daily functioning post-recovery or remission in greater detail.

## Conclusion

This study shows the importance of various symptoms and impacts in individuals with wAIHA. The interviews revealed that fatigue is an important and relevant issue for wAIHA patients, corroborating previously published literature [6, 7]. Patients provided in-depth accounts of fatigue and its associated impacts. The study also identified shortness of breath as an important symptom as it can significantly impact patients’ quality of life, making it essential to explore this symptom more thoroughly in future research and identify PRO measures to assess its severity and impact. The study also provides evidence of content validity and meaningful change for use of the FACIT-Fatigue in measuring fatigue in adults with wAIHA. Recommendations for future studies include evaluation of the psychometric strength of FACIT-Fatigue in the adult wAIHA patient population to provide additional evidence supporting the validity of the measure.

## Supplementary Information

Additional file 1.

## Data Availability

The datasets generated and/or analyzed during the current study are not publicly available due the sensitive nature of the data. Individual participant data or transcripts will not be shared.
