# Supplementary material for: Exploring patients’ experiences with wAIHA and the content validity of the FACIT-fatigue: a qualitative interview study
Source: Orphanet J Rare Dis. 2025 Aug 6;20:403. doi: 10.1186/s13023-025-03767-4 (PMC12326857; doi:10.1186/s13023-025-03767-4)
Supplement: Supplementary file 1 — Additional file 1. [file 13023_2025_3767_MOESM1_ESM.docx]

# Supplementary Tables

**Supplementary Table 1: Patient quotations associated with the signs/symptoms or impacts reported as most bothersome**

| Concept type | Concept reported as most bothersome | Total reports as most bothersome N=19 [n (%)] | Illustrative quotations |
| --- | --- | --- | --- |
| Sign/symptom | Fatigue/tiredness | 18 (94) | “*The fatigue, the tiredness. Because it prevents me from doing regular activities of daily living, like working or making dinner because I’m too tired or doing the laundry because I’m too tired. That’s very bothersome.*” |
|  | Shortness of breath | 9 (47) | “*I wondered if there was something deeper that had been overlooked, because it was not like me to be short of breath or just trying to keep up with the kids and stuff.*” |
|  | Heart palpitations | 8 (42) | “*The heart rate, of course, was bothersome in the sense that you feel like your body’s working harder, so you were just constantly trying to calm your body down. That would just affect everything I was doing. If I noticed my heart was about to beat out of my chest, I just used to stop whatever I was doing and just take a seat or relax. With my heart rate, I couldn’t do a lot of things, even just walking. My doctors told me to get a little bit of exercise. Just walking for an X amount of time would increase my heart rate like crazy. It just affected my daily activities.*” |
|  | Headache | 6 (32) | “*The headaches are very bothersome just because they’re painful and they just go on.*” |
|  | Dizziness | 4 (21) | “*The dizziness is bothersome as well, only because it makes it hard to move*” |
|  | Bodily pain | 3 (16) | “*The pain from the spleen was very much painful. I couldn’t sit down for more than 10 minutes. When I used to a take car ride, I had to lie in the backseat. It was just extremely uncomfortable. I couldn’t sleep. I didn’t sleep on my left side for about 7-8 months because it was enlarged because I would be in so much pain. Even laughing or coughing would cause pain, so that was pretty bothersome.*” |
|  | Treatment-related side effects | 3 (16) | “*The muscle melting. The edema. I would say the hair loss, but that's just vanity. … that bothered me*” |
|  | Skin/eye color change | 2 (11) | “*…the paleness didn’t cause any problems, but it did increase anxiety when I noticed I was really pale.*” |
|  | Mental fatigue/mental tiredness | 2 (11) | “*It just really prevents me from doing stuff because there’s nothing that gets rid of it.*” |
|  | Weight gain | 2 (11) | “*The weight gain is something I’ve never in my entire life had to deal with and now I do a little bit more. Like right now, I’m up about 9 pounds and I started a different exercise program. I actually just started yesterday. But that’s a minor thing in my world because 9 pounds is not going to cause me to have diabetes or anything. I mean, I’m 155 pounds. I’m not big by any means. I really can’t think of a third that’s bothersome or impacted, I should say, if anything.*” |
| Impacts | Feeling anxious or scared | 5 (26%) | “*And then the anxiety coming from… I am still getting blood work done every week. So it’s what is that blood work going to say? Did my levels drop again? What’s the next step? And then looking in the mirror and, if I was pale, being nervous about is it happening all… Am I going to end up back in the hospital? Or with a treatment that I’m really not looking forward to?*” |
|  | Needing help from others | 4 (21%) | “*I mean, here’s something, my daughters were getting older at that time. So they actually, when you say, “Was I getting help?” They were probably helping me out more. I’m pretty sure, and I kind of used it as a way to get them to pitch in more*”  “*I did hate that I had to rely on people, and I still to this day have to rely on people sometimes to help me get through stuff or to stay focused on what needs to be done or whatever.*” |
|  | Impact on work/profession | 3 (16%) | “*Work bothered me that I couldn’t really work the way I wanted to. That’s important to me*” |
|  | Missing time at work | 2 (11%) | “*Work bothered me that I couldn’t really work the way I wanted to. That’s important to me. […] Well, I missed as much. That bothered me.*” |
|  | Impact on social activities | 2 (11%) | “*It has affected my social life in the degree where going out on the weekends*” |
|  | Impact on family life | 2 (11%) | “*Missing out on my kids’ stuff, that’s a big one for me.*” |
|  | Reduced physical strength/mobility | 2 (11%) | “*That I could not be as physically active or productive as I once had been. I don’t know where that would fall. I just didn’t have the energy to be like I once was. That was probably the biggest.*” |
|  | Difficulty with or avoid physical activities | 2 (11%) | “*My inability, I couldn’t do anything.*” |
|  | Impact on leisure activities | 2 (11%) | “*I would say work, career, and travel*” |
|  | Feeling worrisome | 2 (11%) | “*Well, definitely the concern for if it is hereditary, or if… Trying to educate family members. “If you have this combination of symptoms, you need to run, not walk, to your doctor.”*” |
|  | Uncertain about future | 2 (11%) | “*Concern for the future was another one.*” |
|  | Feeling stressed | 2 (11%) | “*But if the Rituxan does work and it is suppressed, just trying to live life and not be…and not let it consume everything. Like I said, I’ve been on the prednisone for so long and I haven’t been in remission yet, but not trying to overthink the symptoms. I think that’s a preoccupation.*” |
|  | Financial impact | 2 (11%) | “*The financial impact because people tend to worry about money and things, and having these constant co-pays, that money could be used for something else. Then you have to figure out what bills you’re going to move around and stuff.*” |

Note: Only those concepts which are reported as most bothersome by at least 2 patients are represented in the table

**Supplementary Table 2: Patient quotation associated with the signs/symptoms reported by at least 5 patients**

| Signs / symptoms** | N=19 [n(%)] | Mean bothersome rating (current)  [n, rating range] | Mean bothersome rating at worst (at diagnosis/flare-up)*  [n, rating range] | Patient quotations |
| --- | --- | --- | --- | --- |
| Dark/brown urine | 12 (63%) | - | 3.0 (n=2; 0 - 6) | - “*My urine started turning a little bit dark. So everybody thought those are signs of dehydration. I started drinking a lot. […] My urine was still constantly during darker, darker, darker..*” - “*It* [Diagnosis] *came about when I was experiencing dark red urine, almost brick red.*” |
| Mental Fatigue/Mental Tiredness | 8 (42%) | 0.0 (n=1) | 6.0 (n=2; 3 - 9) | - “*When the disease was most active, I definitely had a brain fog. I would not describe it as confusion. It was just my thinking was not as clear as it normally was.*” - “*Or that I just couldn’t concentrate or do anything. That was very rare. That was probably when my hemoglobin got to the lowest.*” - “*Difficulty concentrating, kind of like a brain fogginess.*” - “*I think there is a component of my attention span just hasn’t been as focused.*” |
| Appetite loss | 7 (37%) | 2.0 (n=1) | 5.0 (n=1) | - “*That summer I was losing some weight, which is always a good thing for me. I was also…because I also had no appetite.*” - “*Things didn’t taste right. I lost my…certain foods didn’t taste good. I lost my appetite.*” |
| Nausea | 7 (37%) | - | - | - “*I felt like I had a hangover, headache, nausea, and just not feeling good at all, so I stayed home from work.*” - “*I ended up getting really nauseous*” |
| Treatment related side-effects (Signs/ symptoms) | 6 (32%) | - | 8.0 (n=1) | - “*Body swelling, loss of hair, muscle, very, very weak muscles. That's caused by the prednisone and the chemo more than anything else… The symptoms of the treatment were edema, hair fell out, had to pee all the time. Couldn't catch my breath because the diaphragm was so weak… Feeling weak and tired. Oh, I had a steady cough. I was wheezing. And I did get pneumonia during that. I did get pneumonia during the first treatment.*” - “*The prednisone is horrible. I gained a lot of weight on it. Also after being off and on it for so long, it gave me type 2 diabetes, which I didn’t have prior. The wAIHA, the treatments for wAIHA are impacting my health in other negative ways. For example, after the splenectomy, I got a blood clot in my main portal vein. So just having other issues pop up because of it.*” - “*Maybe the stomach pain, I experience it when I was on medications for my treatment for wAIHA… The nausea was more related to when I was getting rituximab. I got 6 rounds of rituximab, so whenever I got a round rituximab, the first, I would say, the first 48-96 hours, I was very much nauseated, so they had to give me nausea medication to help.”* |
| Lightheadedness | 6 (32%) | 0.0 (n=1) | 9.0 (n=1) | - “*I tend to climb stairs to give myself exercise, and so 3 stories…by the time I got on top of 3 stories I’d be like seeing stars.*” - “*Well, lightheaded maybe. Like I said, when I see stars, that would be…that was lightheadedness.*” |

* Bothersomeness ratings were assessed on a 0-10 scale, where 0 = not bothersome at all and 10 = as bothersome as you can imagine.. Average Bothersomeness ratings are based on the number of patients who provided a rating which is not always the same as the number of patients who endorsed the symptom. Some patients provided qualitative descriptions and even with gentle encouragement by the moderator would not provide a quantitative number.** Only symptoms reported by 5 or more patients are shown in the Table. Other signs/symptom included Vomiting (n=4), Muscle weakness/Loss of strength (n=3), Bodily pain (n=3), Fever (n=3), Hear blood pumping (Whooshing sound) (n=3), Muscle pain (n=2), Stomach pains (n=2), and Weight gain (n=2). Other signs/symptoms reported by n=1 patients: Vision loss, Night sweats, Chills/Feeling cold, Loss of consciousness, under eye dark circles, thinning of nails, Constipation, Acid reflux, Weight loss, Pounding in head, and Low blood pressure.

**Supplementary Table 3: Patient quotations associated with the impacts reported by at least 5 patients**

| Impacts** | N=19 [n(%)] | Mean bothersome rating (current) | Patient quotations |
| --- | --- | --- | --- |
| **Emotional impact** | | | |
| Fear of death | 7 (37%) | 10.0 (n=1) | - “*Oh, yeah, there was a night I spent in the hospital, worried I was dying. I think anybody who’s faced with it and this…would have a fear of death.*” - “*Well not sort of like, oh, I’m going to die today. But in general, yeah, it was around. It was like a…it became a presence in my life*.” |
| Uncertain about future | 7 (37%) | 10.0 (n=2; 10 - 10) | - “*Emotionally, because you don’t know if it’s going to come back. Nobody knows why it happened. I mean nobody could give me an answer as to why*.” - “*But now it’s more the uncertainty of if and when I have another relapse or flare up. Because I’ll be good for a while and then something will trigger it. It’s always like, it’s that feeling of when’s the other shoe going to drop. Especially the longer I feel good, the more I’m like, “Uh-oh! When’s it going to happen?” That part is always like, it feels like it’s on the back of my mind a lot*.” |
| Feeling drained | 7 (37%) | 7.0 (n=1) | - “*I’m trying to focus on breathing so I can continue. But focusing on breathing correctly, it drains me. So it’s frustrating that I can’t often find a middle, to where I’m okay to continue to move on.*” - “*Because I was trying to continue to work and I was just so tired*.” |
| Feeling depressed | 6 (32%) | 2.0 (n=1) | - “*I mean, there’s anxiety. With anxiety comes a little depression. I mean, I see a therapist. I take antidepressant... I’m not sure if they’re treating depression or if they’re treating anxiety because it’s the same pills….if I’m in isolation for a long period of time or in treatment for a long period of time, the depression will start setting in a little bit.*” - “*At one point, I thought maybe I’m depressed. Maybe this fatigue, this needing to take all these naps, maybe just not feeling good, maybe it’s…maybe I’m just depressed.*” |
| **Physical functioning** | | | |
| Difficulty walking | 13 (68%) | - | - “*I still have problems walking any length of distance. I would say more than 100-150 feet at a time will cause me to get winded.*” - “*With my heart rate, I couldn’t do a lot of things, even just walking. My doctors told me to get a little bit of exercise. Just walking for an X amount of time would increase my heart rate like crazy.*” |
| Needing frequent rests/Take breaks | 13 (68%) | - | - “*Basically, I would get up and almost as simple as make a cup of coffee have to sit down and take a break. It was totally, totally not like me.*” - “*I’m not much of an exercise person, but I’ll start noticing I’m getting tired throughout the day to the point where I need to start taking naps, or I’m not able to complete a 2-hour stint doing work.*” |
| Difficulty with or avoid physical activities | 9 (47%) | 6.0 (n=2; 4 - 8) | - “*With my son, he likes to play outside or hide and seek in the house. But I can’t be active for too long. So it’s kind of heartbreaking. Now he knows he can’t ask me to play with him. It hurts.*” - “*I can’t exercise. I used to have a Peloton before I was diagnosed. Then once I was diagnosed when I would have flare ups, I was like, “I can’t ride this thing.”*” |
| Trouble getting breath back after physical activity | 8 (42%) | - | - “*I would say the closest that would come to that is I tend to obviously be long-winded, as you might be able to guess. And it’s conceivable that I may have to stop a minute when talking to literally catch my breath because I’ve talked so much that, yeah, I have to stop and catch my breath.*” - “*If you were to do a run, if you went running, your heart rate is higher, your breath is shorter, stuff like that. And as soon as you stop and regroup, within a minute or 2 or 3, you’re back down to a normal level, and that’s pretty much…it was the same thing as that. It was relatively quick.*” |
| Difficulty going down stairs | 5 (26%) | - | - “*The fact that I couldn’t do that was pretty significant, and the fatigue was just like anything I would do, exactly, making the bed or even sometimes going down the stairs or washing a couple of dishes, I’d just be exhausted*” |
| **Activities of daily living/Lifestyle** | | | |
| Impact on leisure activities | 9 (47%) | 5.5 (n=2; 4 - 7) | - “*I’ve had to cancel vacations, I’ve had to cancel different trips because of it. So yeah, it’s frustrating. I get mad.*” - “*Working on like a LEGO model, it’s more difficult to follow directions. I’ll forget things when I’m cooking. Little things like that. Even watching like the news, I’ll lose track of what’s going on unless if I’m really focusing.*” |
| Difficulty with ADLs/Self-care | 7 (37%) | 8.0 (n=1) | - “*The pain from the spleen was very much painful. I couldn’t sit down for more than 10 minutes. When I used to a take car ride, I had to lie in the backseat. It was just extremely uncomfortable.*” - “*I mean, not being able to make dinner or not being able to hang out with friends or go on a cruise, that’s very impactful.*” |
| Difficulty with instrumental activities | 5 (26%) | 9.0 (n=1) | - “*Okay, so during the episodes, my fatigue was pretty bad. When I say that I can’t even make the bed, let’s say if I’m literally trying to pull the... fix the covers on the bed, like just the comforter, that I have to take a minute and just catch my breath.*” |
| **Social impacts** | | | |
| Impact on family life | 6 (32%) | 8.0 (n=2; 7 - 9) | - “*It affects family life because I don’t have the energy to participate in as many activities as maybe the rest of the family.*” - “*Often, I would say severe to where I can’t often do family functions. They basically stopped inviting me because they already know I most likely won’t come.*” |
| **Professional impacts** | | | |
| Impact on work/profession | 8 (42%) | 7.7 (n=3; 6 - 9) | - “*I’ll just notice that it’s taking me a lot longer to do my work...But I will have to do the same task 2 or 3 times to complete it because I’ll read the same things over and over, and I won’t be able to fully comprehend what I just read.*” - “*I wasn’t able to put in as much time or as much mental focus as I had before. I didn’t have the energy to sustain a normal day.*” |
| **Other impacts** | | | |
| Impact on sleep | 9 (47%) | - | - “*There are some nights I can’t sleep because it feels like my heart is just going to thump itself out of my chest.*” - “*A lot of that is because my whole sleep pattern is completely thrown off. When I take a nap or two naps a day, then my evening schedule is off, my nighttime sleep is off, and so it’s a vicious cycle.*” |
| Financial impact | 7 (37%) | 8.0 (n=2; 8 - 8) | - “*It has affected me financially. I had medical bills that I had to pay and had to pay someone to keep up my farm and stuff while I was sick.*” - “*Financially, the medical bills are insane. I do have really good insurance. But even the copays, all the diagnostic tests I had, all the hospitalizations I’ve had for the blood transfusions, they’re just astronomical.*” |
| Treatment related side-effects (Impacts) | 6 (32%) | 8.0 (n=1) | - “*You know when you're taking pain meds, I'm taking oxy right now, that changes your whole outlook on absolutely everything. It changes your appetite. It changes your mental mood.*” - “*Everything was very annoying. Even the dogs… I love my dogs. Even the dogs were annoying. I'd want to just go and just leave me alone, let me play on my phone, let me play a game on my phone. I'd be so into that because the prednisone would make me so hyper focused and so awake.*” |

* Bothersomeness ratings were assessed on a 0-10 scale, where 0 = not bothersome at all and 10 = as bothersome as you can imagine. Average Bothersomeness ratings are based on the number of patients who provided a rating which is not always the same as the number of patients who endorsed the impact. Some patients provided qualitative descriptions and even with gentle encouragement by the moderator would not provide a quantitative number. ** Only impacts reported by 5 or more patients are shown in the Table. Other emotional impacts included impact on overall mental state (n=4), feeling stressed (n=3), feelings of panic (n=2), lack of motivation (n=1), embarrassment (n=1), feeling guilt (n=1). Other physical functioning impacts included difficulty talking (feeling breathless) (n=4) and need to slow down (n=3). Other activities of daily living/lifestyle impacts includes unable to drive/difficulty driving (n=2). Other social impacts includes not able to go out (n=3. Other impacts includes need for frequent or pre-planning medical appointments (n=3).

**Supplementary Table 4: Patient feedback for the FACIT-Fatigue on item relevance to their experience of wAIHA, item clarity and comprehension**

| Item | # Patients able to answer | # Patients to whom item relevant | # Patients understood item* | Patient-provided reasons for irrelevance/difficult to understand/Suggested changes (if appropriate) |
| --- | --- | --- | --- | --- |
| FACIT-Fatigue  Instructions | N/A | N/A | Yes = 18;  Yes, with difficulty = 1 | **Understood with difficulty:**   - "*I think the only unclear thing would be the “circle or mark”. I think having one or the other would be more clear because, I mean, I see there’s boxes, so I would assume putting an X or a checkmark in the box rather than circle. I wouldn’t necessarily know, do you want me to circle the number 0 or the “not at all”.*" |
| FACIT-Fatigue  Item 1 –  I feel fatigued | Yes = 19 | Relevant = 15;  Not relevant = 1;  Not asked = 3 | Yes = 18;  Yes, with difficulty = 1 | **Understood with difficulty:**   - **“*Is there anything confusing there?*** *Well, yeah. Like we talked about earlier, how you break out fatigue, weakness. Fatigue is how I feel. Weakness is a manifestation of how my muscles are working.*”   **Suggested changes:**   - **Overlap with other items:**   - "*I mean, the questions seem a little redundant sometimes. That's not to criticize you directly or whoever is the maker of this, but I mean, you can capture “I feel fatigued” or “I feel like I have no energy at all”. “Washed out” seems definitely the same as “I need to sleep all the time.” in my opinion. Or it could refer to “I emotionally feel incapable of doing…” I don't know. That's how I would probably try to word it, if that makes sense.*"   - "*Well, it's [Item #4] the same as “fatigued” to me. So I would maybe replace “fatigued” with “tired”.*"   - "*I mean, it could be better with more…specifically not to use the literary definition of fatigued or washed out or tired, but almost they’re the same questions, in my opinion. So if you’re really…if the goal really is to measure each thing individually, I guess more examples for each one would be better.*"   - "*Giving more guidance, like on what does this mean to feel fatigued? What are you trying to get at with “fatigued”? What are you trying to get at with these 4 dimensions here that differentiate them? Rather than me just reading it and how do I…what do I just think at the moment about it. Is it okay if I think they’re all the same? I would be trying to figure out are they really trying to get something different. What is that? More of a puzzle.*"   **Not relevant:**   - “*This is not a fair representation as to how wAIHA is making me feel right now*” |
| FACIT-Fatigue  Item 2-  I feel weak all over | Yes = 18; Not asked = 1 | Relevant = 12;  Not relevant = 1;  No, but relevant for others = 2;  Not asked = 4 | Yes = 17;  Yes, with difficulty = 2 | **Understood with difficulty:**   - "*“I feel weak all over.” Initially I’d say, is that muscle weakness? I consider that like a physical ability. It’s similar to fatigued, but I can make a clarification. I feel weak. I don’t necessarily know that it’s a good description. In my view, I feel weak.*" - "*Because “weak all over” I would think is, I don’t know, is it like physical, mental? It’s kind of confusing to me.*"   **Suggested changes:**   - **Overlap with other items:**   - "*Well, I wouldn't put it in there. I'd just leave it. I just leave it as 1 and not do 2.*" - **Change in terminology:**   - "*Giving more guidance, like on what does this mean to feel fatigued? What are you trying to get at with “fatigued”? What are you trying to get at with these 4 dimensions here that differentiate them? Rather than me just reading it and how do I…what do I just think at the moment about it. Is it okay if I think they’re all the same? I would be trying to figure out are they really trying to get something different. What is that? More of a puzzle.*"   - "*I think I would like a little bit more clarification regarding is this like a physical weakness or it’s just like a general system weakness, but I’m going to interpret it as physical.*"   - "*I don’t know, maybe adding the word...like depending on what you guys are looking for...maybe like “physically”, “I feel weak physically.”*"   **Not relevant:**   - "*It is not important to my experience. I’m like I don’t get this type of a symptom, but I think it’s important to help differentiate between “fatigued and weakness” because I think those for a medical professional might be a good differentiation of 2 different things.*" |
| FACIT-Fatigue  Item 3 –  I feel listless (washed out) | Yes = 18; Not asked = 1 | Relevant = 18;  Not asked = 1;  Less relevant than item #1 & #2 = 1 | Yes = 12;  Yes, with difficulty = 6;  No = 1 | **Not understood:**   - "*I have no idea what that means. I guess maybe burnt out. I mean, I get the feeling that they’re trying to go for mental fatigue there. I guess I just feel blah, but it’s kind of confusing. I’m not exactly sure what they’re looking for […] It doesn’t help for me. I think a better example of it would be better, okay. [...] Yeah, I’m not sure if they’re looking for like emotional here, or are they looking for mental. Yeah, I’m not sure what they’re looking for.*"   **Understood with difficulty:**   - “*That one’s a little unclear. Yeah. I mean, I understand it. Like, “I feel like nothing, like jelly.” It's another way of saying, “I feel fatigued,” in my opinion.*" - "*Like people will say “You look washed out,” meaning that you look pale. I think that could be confusing.*" - "*Well, I feel like people can have a different understanding of the term “washed out,” so I feel like this could be made more clear. Like maybe with examples.*" - "*Because to me, when I see washed out, it makes me think of physical appearance almost. We talked about pale and jaundice, to me at least the word listless feels more like… What’s the word? Let me think of the word I’m looking for. Listless is like unable to settle down almost. Maybe I’m reading it wrong, but that #3 doesn’t make a lot of sense to me.*" - "*I kind of feel like that. It's kind of a confusing one for me.*" - "*Because I don’t know if they want me to answer emotionally or physically*"   **Suggested changes:**   - **Overlap with other items:**   - "*I mean, I would probably just get rid of it. It feels very redundant…I mean, the questions seem a little redundant sometimes. That's not to criticize you directly or whoever is the maker of this, but I mean, you can capture “I feel fatigued” or “I feel like I have no energy at all”. “Washed out” seems definitely the same as “I need to sleep all the time.” in my opinion. Or it could refer to “I emotionally feel incapable of doing…” I don't know. That's how I would probably try to word it, if that makes sense.*" - **Change in terminology:**   - "*I would say not “washed out” but “worn out”. “I feel listless or worn out.”*"   - "*I really just have an issue with the whole “washed out” thing. I really feel like I need a better understanding in general of, I guess, maybe what other people have considered to be washed out.*"   - "*Maybe aimless, unable to settle down, something like that. Yeah. Because I get the feeling of listlessness. It’s like when you kind of don’t know what to do with yourself and you’re tired, but you don’t want to sleep. I guess I can kind of explain it, but I’m not sure. Well, I would need a whole paragraph…But I would take out the washed out part in parentheses. Because to me, washed out is more either fatigue or washed out in terms of how you look physically.*"   - "*I just think it needs to be rephrased a little bit.*" |
| FACIT-Fatigue  Item 4 –  I feel tired | Yes = 18; Not asked = 1 | Relevant = 15;  Not relevant = 1;  Not asked = 3;  Less relevant than item #1, #2 & #3 = 1 | Yes = 19 | **Suggested changes:**   - **Overlap with other items:**   - “*Yeah, I guess “tired”, not sure I would necessarily see a difference between “tired” and “listless” if I had to fill this out. But if you forced me to, to say they are all intended to be different, I would say… I would actually say “tired” was closer to “fatigued”, that “listless” was like the least impactful.*”   - "*If I picked weak, listless, tired. “Tired” is just too redundant. So you might be able to…Yeah. Just go with those 3, a little less confusing.*"   - "*That question is a little teeny bit redundant to me as “I feel fatigued”. Because they kind of… This question might want to be the first question. Because you’re kind of like going down, tired, fatigued, weak all over, washed out. To me, tired is the least of all of those.*"   - "*Well, it's the same as “fatigued” to me. So I would maybe replace “fatigued” with “tired”.*"   **Not relevant:**   - "*Because it really…to me, it doesn’t really pinpoint a symptom as far as the wAIHA. Because everyone gets tired, from what I explained I think tired is.*" |
| FACIT-Fatigue  Item 5 –  I have trouble starting things | Yes = 18; Not asked = 1 | Relevant = 17;  Not asked = 2 | Yes = 18;  Yes, with difficulty = 1 | **Understood with difficulty:**   - **“*Do you think this question is clear?*** *No, I don’t.*”   **Suggested changes:**   - "*I think it would be clearer, “I have trouble starting and ending things because I am tired.”*" |
| FACIT-Fatigue  Item 6 –  I have trouble finishing things | Yes = 18; Not asked = 1 | Relevant = 16;  Not relevant = 1;  Not asked = 2 | Yes = 19 | **Not relevant:**   - "*I feel bad, but I'm going to have to say that one's irrelevant to me, but once again, that's because I…what I said about I wouldn't start it.*" |
| FACIT-Fatigue  Item 7 –  I have energy | Yes = 17; Not asked = 2 | Relevant = 15;  Not relevant = 3;  Not asked = 1;  Less relevant than item #8 = 1 | Yes = 13;  Yes, with difficulty = 6 | **Understood with difficulty:**   - "*Yeah, I think it is [ambiguous]. You can do that with #3.*" - "*It doesn’t give a clear indication, like if it means do I have energy overall or energy to finish a task or energy to finish what I start.*" - "*It sounds like a really bad business slogan...This one’s a little...it seems like it’s missing the rest of the sentence. “I have enough energy. I have sufficient energy.”*" - "*My brain was always going, my body wasn’t. Energy to me is relative. I would think of “I have physical energy” versus mental energy would help me on that question.*" - "*I think if they’re wanting to know, “Are you back to your baseline level of energy?” they might want to clarify it.*"   **Suggested changes:**   - **Overlap with other items:**   - "*I don't think you need #8 [sic. #7]. That's ambiguous. I mean I have energy, but how much energy? What can I do with that energy? I have energy to turn on the television set, but I don't have energy to finish watching the show...I feel pretty strongly about #8 [sic. #7]. I feel strongly about #8 [sic. #7]. I don't like this one.*"   - "*I mean, the “I have energy” is…well, I don’t know. But that seems like it should have been first maybe. I don’t know why. It’s just, it seems like more of an overarching thing it’s like. But it’s fine.*"   - "*I think it’s more with the item itself. I think there needs to be a delineation about the energy. I have enough energy. I have energy to do my daily work. It just seems like there needs to be more, if that makes sense.*" - **Changes in terminology:**   - "*You can just ask, “Have energy to finish all tasks”.*"   - "*I would think of “I have physical energy” versus mental energy would help me on that question.*"   **Not relevant:**   - "*Just because of the…maybe because it’s so minimal and with the diagnosis, it’s not something I’ve experienced a lot of.*" - "*I don’t know that it is very important from my experience. I think some of the other questions are much better suited than this one would be for me.*" |
| FACIT-Fatigue  Item 8 –  I am able to do my usual activities | Yes = 18; Not asked = 1 | Relevant = 19 | Yes = 18;  Yes, with difficulty = 1 | **Understood with difficulty:**   - “*When you say usual activities, can I shower? Yes, I can shower. Can I brush my teeth? Yes, I can brush my teeth. But if you’re talking about anything more like, can I make dinner? No, I can’t. Can I vacuum? No, I can’t do that either. I think it depends on what kind of activities.*”   **Suggested changes:**   - "*Well, you might want to put in like “Getting dressed and cooking breakfast” or something.*" |
| FACIT-Fatigue  Item 9 –  I need to sleep during the day | Yes = 18; Not asked = 1 | Relevant = 17;  Not relevant = 1;  Not asked = 1 | Yes = 17;  Yes, with difficulty = 2 | **Understood with difficulty:**   - "*So I have a little question mark in my head because I’m like, well if I…because sometimes if you’re anxious or whatever, you don’t sleep as well. And then you… For me, I mean, I would need…if I wake up at 5:30 in the morning, I need a nap during the day. Versus a day where I just got a regular 7-1/2 hours of sleep or whatever. And then if I still needed to sleep during the day, so that’s a little confusing.*" - “***Do you feel that this question was clear?*** *No. I don’t think it’s clear.[…] Because the actual need to sleep, if you really need to sleep, your body is just going to go sleep. You’re just going to fall asleep at work. You’re going to fall asleep driving. I think “I feel the need” or “I want to sleep” is a little more closer to what we experience.*”   **Suggested changes:**   - "*I mean, the questions seem a little redundant sometimes. That's not to criticize you directly or whoever is the maker of this, but I mean, you can capture “I feel fatigued” or “I feel like I have no energy at all”. “Washed out” seems definitely the same as “I need to sleep all the time.” in my opinion. Or it could refer to “I emotionally feel incapable of doing…” I don't know. That's how I would probably try to word it, if that makes sense.*" - "*The only way that this could be an issue is if there’s somebody who works third shift because they may be sleeping during the day already.*" |
| FACIT-Fatigue  Item 10 –  I am too tired to eat | Yes = 18; Not asked = 1 | Relevant = 10;  Not relevant = 3;  No, but relevant for others = 2;  Not sure = 2;  Not asked = 2 | Yes = 18;  Yes, with difficulty = 1 | **Understood with difficulty:**   - "*I mean, too tired to eat would mean…there again, I’m like, okay, does that mean I’m too tired to get up and get some food together and put it on a plate and go sit at the* *table and eat it? Or does it mean I’m too tired even the candy bar I have in my hand right now, to eat that or the whatever snack thing that I…that is right here, I can’t even eat it. Or maybe you feel your tiredness makes you feel nauseous or… I don’t know. There’s a bunch of things that fall under there.*"   **Suggested changes:**   - "*I just think you need to clarify #10 what you’re really asking, the “I’m too tired to eat”. I’m too tired to get…to prepare food or I’m with food in front of me, I’m too tired to even eat it.*"   **Not relevant:**   - "*I have never run into this problem. Well...no, I’ve never run into this problem personally, so for me, it’s “not at all”. It hasn’t been my experience, so I don’t know how applicable it might be to other people, but it’s not important for me.*" |
| FACIT-Fatigue  Item 11 –  I need help doing my usual activities | Yes = 18; Not asked = 1 | Relevant = 16;  No, but relevant for others = 2;  Not asked = 1 | Yes = 19 | **Suggested changes:**   - "[Clarification] *On what the activities* [like hygiene, self-care]" |
| FACIT-Fatigue  Item 12 –  I am frustrated by being too tired | Yes = 18; Not asked = 1 | Relevant = 16;  No, but relevant for others = 1;  Not asked = 2 | Yes = 19 | **Suggested changes:**   - "*I don’t know how to suggest it, but like I said, #12 and #13 are very similar, in my mind.*" - PID001 - "*I think fatigued would be the term I would use versus tired. Tired feels like something you can fix by taking a nap. Fatigue is something that cannot be…it’s just there. So with my experience, I felt more frustration with fatigue and just overall low energy versus tiredness.*" |
| FACIT-Fatigue  Item 13 –  I have to limit my social activity because I am tired | Yes = 17; Not asked = 2 | Relevant = 18;  Not relevant = 1 | Yes = 19 | **Suggested changes:**   - "*I don’t know how to suggest it, but like I said, #12 and #13 are very similar, in my mind.*" - "*I mean, I guess you can define social activity a little more. To say are you talking very specifically like shopping versus going out and playing beach volleyball?*"   **Not relevant:**   - "*I have never ever limited social activity because I'm tired. I'll go there and fall asleep if I have to, but I'm not missing out on social activity.*" |

**Supplementary Table 5: Patient feedback for the FACIT-Fatigue response option clarity and comprehension^^[[1]](#footnote-1)^^**

| Response option | # Patients to whom response options is clear | # Patients to whom provided their interpretation of each response option [n(%)] | Patient description of the response option |
| --- | --- | --- | --- |
| FACIT-Fatigue  Item 1 –  I feel fatigued | Clear = 18; Not asked = 1 | Not at all - 11 (58%) | - “*Feeling good, well rested”* - Have *“normal level of energy”* - *“Wouldn’t be taking naps during the day”* - Feeling *“great every day from the past 7 days”* |
|  |  | A little bit - 12 (63%) | - *“Couple of times in a week”*, once a day, *“a day or a day-and-a-half”* - “*10-20%*”, “*25%*” of the time, - Feeling “*it's* [Fatigue] *still there but just not bothering you very much*” - “*Having to take a few extra breaks throughout the day*” |
|  |  | Somewhat - 15 (79%) | - “*Sometimes I'm tired but it's not the worst*” - Not feeling great “*3 times a week*” or “*50% of the time*” - Skipping an activity which would have been done in normal circumstance - Needing “*a little bit of a nap a day*” - Not having the energy to accomplish all of the daily tasks |
|  |  | Quite a bit - 16 (84%) | - “*Prevalent*” fatigue which is occurring “*every couple of days*”, “*60-70%*” of the time or “*5 days out of the week*” - Bothering “*quite a bit but not all the time*” - “*Periods of transient fatigue*” - Constantly trying to recharge energy - Needing 15 or 20-minute nap every day |
|  |  | Very much - 15 (79%) | - Feeling fatigued “*consistently*”, “*every day*” or “*6-1/2 to 7*” days - Bothering a lot - “*Can barely get out of bed*” - Unable to do daily activities - Need naps |

**Supplementary Table 6: Patients’ assessment of perceived meaningful change on the FACIT-Fatigue Item 1 (I feel fatigued)**

| Response option | Number of patients who indicated that they would need a  minimum meaningful change of: | | | | | | |
| --- | --- | --- | --- | --- | --- | --- | --- |
|  | N | Improvement (N=19) | | | Worsening (n=16) | | |
|  |  | -1-Point | -2-Point | -3(+)-Point | +1-Point | +2-Point | +3(+)-Point |
| 0 (not at all) | 5 | 3 | 2 | 0 | 4 | 0 | 0 |
| 1 (a little bit) | 6 | 5 | 1 | 0 | 4 | 2 | 0 |
| 2 (somewhat) | 3 | 3 | 0 | 0 | 2 | 1 | 0 |
| 3 (quite a bit) | 2 | 1 | 1 | 0 | 2 | 0 | 0 |
| 4 (very much) | 3 | 3 | 0 | 0 | 1 | 0 | 0 |
| Total (%) | 19 | 15 (79%) | 4 (21%) | 0 (0%) | 13 (81%) | 3 (19%) | 0 (0%) |

1. The relevance and clarity was only tested on item 1 of the FACIT-Fatigue as the response options were same for all items [↑](#footnote-ref-1)
